# A German Model Project for Workplace Health Promotion—Flow of Communication, Information, and Reasons for Non-Participation in the Offered Measures

**DOI:** 10.3390/ijerph19138122

**Published:** 2022-07-01

**Authors:** Regina Lutz, Wolfgang Fischmann, Hans Drexler, Elisabeth Nöhammer

**Affiliations:** 1 Institute and Outpatient Clinic of Occupational, Social and Environmental Medicine, Friedrich-Alexander-Universität Erlangen-Nürnberg (FAU), Henkestraße 9-11, 91054 Erlangen, Germany; wolfgang.fischmann@fau.de (W.F.); hans.drexler@fau.de (H.D.); 2 Department for Public Health, Health Services Research and HTA, UMIT—Private University for Health Sciences, Medical Informatics and Technology, 6060 Hall in Tirol, Austria; elisabeth.noehammer@umit-tirol.at

**Keywords:** health promotion, occupational health, communication, dissemination, information, microenterprises, small businesses, medium-sized businesses, Germany

## Abstract

Background: Workplace health promotion (WHP) as a part of workplace health management (WHM) was strengthened in German legislature with the Prevention Act of 2015. However, smaller enterprises often do not offer WHM or WHP. Accordingly, a model-project for improving the uptake and implementation, particularly in micro-, small, and medium-sized enterprises (MSMEs) was carried out. The aim of the study was to determine reasons for non-participation in WHP offers and analyze communication issues, both from the employee’s and employer’s perspective. Methods: In total, 21 managers or persons responsible for WHP participated in the first online survey between March and April 2020, and 156 employees responded to the second online survey between June and October 2021. The importance of barriers and communication issues was investigated. Based on a principal component analysis on non-participation, differences regarding sociodemographic variables were analyzed. Results: Most employees knew about the offered measures and that the measures were cost free. There was no significant association between having communicated the offers to the employees and considering them suitable for their needs. Most of the managers or persons responsible for WHP rated the measures as sufficiently varied and allowed staff to take part during working-time. Reasons for non-participation from the managers’ point of view were travel time to the location of the offers, lack of time, and a missing fit between offers and employees’ needs. From the employees’ point of view, workload (including working time) was the main barrier to participation. Conclusions: For the practical implementation of model projects in MSMEs, special attention should be paid to ensuring opportunity to participate, which may be easier with in-house offers.

## 1. Introduction

It is important for companies to have healthy employees. In addition to fulfilling the legal requirements of occupational safety and health (OSH) and being relevant to the success of an organization, healthy employees are also a prerequisite for innovation, progress, and growth [1,2]. From an occupational (medical) and psychological point of view, it is of great importance to attain and improve physical and mental health [3]. Shift work, work stress, and work demands are correlated with the health and well-being of employees [4,5,6]. Lost working days, increasing absenteeism, and presentism with a reduced productivity can be a consequence [7]. In addition, this is of financial relevance. In 2020, most days of inability to work in Germany were caused by mental illnesses [8,9,10]. In this context, not only direct illness costs but also indirect costs caused by production losses must be considered [11,12]. Being of individual, organizational, and societal relevance, maintaining health is a shared responsibility of employees and the health-oriented behavior of managers in company practice [13,14,15,16,17], based on and extending legal requirements. Thus, many employers aim to implement workplace health management (WHM) [18]. WHM supports the establishment, integration, and the management of healthy working conditions in companies or organizations [19,20]. The aim is to promote the health, performance, and employability of employees in the workplace setting [20]. In addition to OSH measures and a company reintegration management program after long illnesses of individual employees (mandatory by law in Germany), WHM consists of the subareas of workplace health promotion (WHP) and supporting personnel development (both voluntary) [20]. WHP can also be compared with the “Total Worker Health” concept implemented in the USA, which has been developed over the last 20 years [21].

WHP as a part of WHM was strengthened in German legislature with the Prevention Act of 2015 (PrävG), which included the workplace setting [22]. Based on a joint national prevention strategy, not only municipalities, day care centers, schools, and care facilities but also companies are invited to fulfill and cooperate in tasks of health promotion [22]. According to article 20g SGB V PrävG, it is possible to carry out model projects in the settings mentioned. This is intended to improve the quality and efficiency of benefits for WHP and prevention [22]. One such model project to improve acceptance and implementation, especially in MSMEs, was carried out in Thuringia, Germany (see Section 2.1).

Many companies offer measures of WHP to their employees [23], but the participation of the latter is often low [24,25,26,27,28,29]. To improve the success of an intervention, implementation science and change management provide various suggestions and models. Since it focuses on the individual and includes intervention and policy areas, the behavioral wheel is one model of specific relevance in this context. The basic premise is that behavior (change) is dependent on capability, motivation, and opportunity [30]. This underlines that a combination of individual and setting-related aspects must be covered to increase the likelihood of WHP participation (for an overview, see e.g., [31,32,33]). Moreover, research shows that the implementation of WHM or fulfilling legal requirements of OSH is often related to company size. WHM or OSH measures are more frequently implemented in larger companies [34,35,36,37,38]. It can, therefore, be assumed that, in structurally weak or rather rural regions, a WHM or OSH is not fully implemented [34], making model projects advisable. In addition to structural relevance, these can provide valuable insights regarding barriers to WHP implementation and utilization. Besides the above-mentioned studies, literature on WHP networks in MSMEs is scarce [39]. Thus, the present analysis can provide further insights for this subarea.

In this study and as part of the model project (see Section 2.1), evaluations of the program and the offers were carried out among (WHP-)managers as well as employees. The aim was to determine attitudes toward WHP and to identify reasons for any non-participation in WHP offers.

We considered the following aspects as part of the study:

1. To analyze the flow of communication and information about WHP offers from company management to employees.
2. To investigate the support of managers or persons responsible for WHP and to present the reasons for non-participation of the employees from the managers’ point of view.
3. To present the reasons for non-participation of the employees from the employees’ point of view.

In the course of the project, the topic of barriers became prominent, so they were investigated in more detail in a stepwise manner to approximate the issue as best possible. The descriptive analysis of these aspects provides insights not only into the potentials of WHP network projects but also into their limitations, which need to be tested in subsequent research. Special attention is paid to the differences in the perspectives of the employees and the managers or those responsible for WHP. Recommendations for achieving higher participation rates in the future are discussed, based on the results. 

## 2. Materials and Methods

### 2.1. Model Project and WHP Offers

In 2017, the model project “Gesund arbeiten in Thüringen (GAIT)” (Healthy working in Thuringia) was founded and launched. The project, which is in accordance with article 20g SBG V PrävG, focuses on improving WHM and WHP uptake and implementation, particularly in micro-, small, and medium-sized enterprises (MSMEs) in rural and structurally weaker regions. The project was set up in Thuringia, a structurally weaker region, as a cooperation of BARMER health insurance as financing partner, German Association of Occupational Medicine and Environmental Medicine (Deutsche Gesellschaft für Arbeitsmedizin und Umweltmedizin (DGAUM)) as managing partner and several scientific institutions, with Friedrich-Alexander-Universität Erlangen-Nürnberg (FAU) being responsible for network issues.

With the aim of providing employees and companies in a better and sustainable manner with occupational medicine services, to strengthen OSH in companies and to promote exchange and support between companies, three regional company networks were founded as part of the project (one each in central, southern, and eastern Thuringia). These consisted of a total of 30 MSME companies in the service, trade, health and social services, and manufacturing sectors. 

To support the network companies in OSH and to comply with the legal requirements for risk assessment (article 5 ArbSchG), an initial survey of the employees was offered to all network companies. The survey was designed in a way to also include employees’ perceived WHP demands and needs. Based on the survey results of those network companies that participated in the survey, WHP measures were designed. These were financed by BARMER health insurance, offered between January 2020 and December 2021 at specific easy to reach locations plus virtually (especially at the beginning of the SARS-CoV-2 pandemic), and could be used cost free by the companies’ employees. The measures covered behavioral and setting-related prevention: in addition to workshops for managers in all three networks, offers in the areas of team building, physical activity, nutrition, and stress management were provided in all three network regions. The digital measures were partly offers via specific apps (for further information to the interested reader regarding the initial survey, see [40]).

Basing the offers on a survey of employee needs and interests regarding WHP can help to cover the motivation aspect as well as their capabilities. The latter were also considered by providing a broad range of measures with different degrees of complexity. While, e.g., travel time and accessibility were taken into account by the model project management when choosing the locations for the offers, safeguarding that these could be reached in a reasonable time by employees of all network companies, providing the opportunity for WHP offer use, however, also depends on the efforts of the companies. Most importantly, employees need to be informed about the offer and given the permission and possibility to participate [30,41]. 

To initiate the flow of information and communication in the model project, managers or persons responsible for WHP (a) were sent e-mails with more information regarding the offered measures and (b) an online platform for communication across the network companies was provided. In network company meetings, opportunities for employees to participate in the offered WHP measures were discussed, and suggestions were made as to how the offers could be communicated (e.g., personally, via team meetings, or by e-mails) and how they could be made accessible to employees (e.g., allowing participation during working time, to providing means of transportation).

### 2.2. First Survey (Managers and Persons Responsible for WHP in the Network Companies)

Managers and those responsible for WHP in the network companies were invited to participate in an online survey via LimeSurvey between March and April 2020. Accordingly, inclusion criteria were as follows: participants had to be managers or those responsible for WHP in the network companies of the model project GAIT and German speaking. All others were excluded. Beside sociodemographic questions such as sex, the position in the company, and items on the company itself, information and communication of the model project and the participation or non-participation of the employees (four open and ten closed items) were asked. One week after the first invitation e-mail, a reminder was sent. The respondents were informed that study participation was voluntary, and that the data would be analyzed and published anonymously. Due to that and the types of questions asked, no ethical approval of the study was required according to the ethical standards of FAU. 

### 2.3. Second Survey (Employees)

As part of the above-mentioned project, the 30 network companies were offered a full employee survey (see Section 2.1) in the period from June to October 2021. The survey was also conducted as an online survey via the LimeSurvey platform. Participation was voluntary for both companies and staff. To generate the broadest possible database, all employees (at least 18 years old and German speaking) of the network companies were invited to participate. No restrictions were placed regarding work experience or working hours. Exclusion criteria were age below 18, non-German speaking, and not being employed in one of the network companies. The employees received universal links that were created individually for each participating company, and taking part was anonymous. The results were aggregated to ensure that the most detailed level of analysis was the (entire) company. 

In addition to sociodemographic items such as age, sex, and position in the company, the survey included items on the flexibility of work, communication of the project, communication regarding the WHP measures and questions about participation or non-participation in the offered WHP measures. 

Project and WHP-measure communication were surveyed using three closed questions with nominal characteristics (“yes”, “no”, “I don’t know”, plus the option of “no response”) and two open questions. The topic area for non-participation in the WHP measures was based on Nöhammer et al. [29] and consisted of 15 closed items with an ordinal four-point Likert scale (“applies” (1), “does rather apply” (2), “does rather not apply” (3), “does not apply” (4); plus, the option of “no response”).

### 2.4. Data Analysis

All statistical analyses were performed with IBM SPSS Statistics (versions 21 and 28). Frequencies for the first and second survey were calculated descriptively. Additionally, an existing relationship between the communication (“Were the measures communicated?”) and the assessment of managers or persons responsible for WHP (“The WHP offers do not fit the needs of our workforce.”) was checked via Fisher’s test. For the above-mentioned 15 items for non-participation in WHP offers as part of the employee survey, principal component analysis (exploratory factor analysis, EFA) with varimax rotation was performed to reduce them to a lower number of underlying dimensions [42]. For assessing the suitability of the 15 items for EFA the Kaiser–Meyer–Olkin criterion (KMO) was used. A KMO value of 0.6 or higher confirms suitability of the items (high correlations) [42]. The factor loadings had to be at least 0.4, and the factor extraction was performed with an initial eigenvalue >1 for a factor to be extracted [43,44]. As a result, sum scores were calculated for the remaining dimensions, as well as reliability measures (Cronbach’s alpha). Moreover, Kendall rank correlations were performed for testing relationships between the dimensions of non-participation and age of the employees. For differences in sex regarding non-participation, *t*-test, and Mann–Whitney U-test were used.

## 3. Results

All surveys were done in German, the items and responses stated were translated to English by the authors. 

### 3.1. Participants and Sample Characteristics

Table 1 displays the sociodemographic characteristics of the two surveys. In the following, “employees” is (again) used as term comprising all participants of this survey. We use the term differently (and with “regular” as addition) only in this table to provide information on the position in the organization. 

In the first survey, we did not ask the age of the managers or persons responsible for WHP because of no relevance to the topic. Table 2 shows the response rates of both surveys. The column of the second survey shows, on the one hand, the response rates by company and, on the other hand, the responses depending on the total number of employees.

### 3.2. Flow of Communication and Information

In total, 76.2% (*n* = 16 of 21) of managers or persons with responsibility for WHP replied that they had communicated the offers to the employees (first survey). In the survey of the employees (second survey; *n* = 156), 51.9% (*n* = 81) stated they were informed about the interventions taking place, 25% (*n* = 39) stated not having been informed, and 16% (*n* = 25) could not remember. Considering those network companies in which the managers or persons responsible for WHP stated that the measures were communicated (first survey), 63.6% of the employees (*n* = 56 of 88) were informed, 17% (*n* = 15 of 88) were not informed, and 14.8% (*n* = 13 of 88) stated that they did not know anything about measures (second survey).

Regarding the flow of information that participation in WHP offers would be cost free, 58.3% (*n* = 91 of 156) of the employees responded they did know, while 38.5% (*n* = 60 of 156) did not know about that fact. In the network companies where the managers or persons responsible for WHP communicated the measures, the proportion of those who knew that the measures were cost free was higher 70.5% (*n* = 62 of 88), as only 25% (*n* = 22 of 88) replied they had not known.

Those managers or persons responsible for WHP who communicated the measures (*n* = 16 of 21) were asked whether the employees were reminded after the first communication. To this, 81.3% (*n* = 13 of 16) responded that the employees had been reminded of the measures (by the managers or persons responsible for WHP themselves or another person), while 18.7% (*n* = 3) did not remind them. Reasons for not reminding were that the employees of one company “attended the measures immediately” and “the Corona pandemic” for another company. 

The managers or persons responsible for WHP who did not communicate the measures to the employees were asked for the reasons for not doing so. The answers to this open question were very divergent: It was reported that the measures were not targeted at their employees’ needs, that WHP was currently no issue, that the current focus is on the reorganization of the company, and one person responded that they needed to take a closer look at the measures. Fisher’s test on the relationship between the communication of offers to the employees (yes/no) and whether the WHP-offers did (not) fit the needs of the companies’ workforce showed no significant correlation (*p* = 0.49).

### 3.3. Acceptance of Interventions and Reasons for Non-Participation from the Mangers’ Point of View or According to Those Responsible for WHP

To analyze the attitudes of the company management toward the offers, it was asked whether the range of measures within the four areas of WHP (physical activity, nutrition, stress management, and addiction prevention) is perceived as sufficient, whether cross-network offers are welcome, and whether there are suggestions for improvement regarding the offers. We found that 35% (*n* = 7 of 20) agreed strongly in rating the offers as sufficiently varied, 50% (*n* = 10 of 20) agreed, 15% (*n* = 3 of 20) disagreed, and no one strongly disagreed. Moreover, 45% (*n* = 9 of 20) agreed strongly that cross-network offers are welcome, 40% (*n* = 8 of 20) agreed, 15% (*n* = 3 of 20) disagreed, and no one strongly disagreed. Suggestions for improvements were to better consider small enterprises with a high workload, that measures should be offered close to the company (maximum 30 min driving time), and that the organizer should not communicate the measures last minute.

The managers or persons responsible for WHP were additionally asked whether their employees took part in the measures. To this, 40% (*n* = 8 of 20) responded that the employees took part at least once or more often, 55% (*n* = 11 of 20) stated that the employees had not participated yet, and 5% (*n* = 1 of 20) did not know.

Afterward, the 11 persons who had responded that the employees had not yet taken part in measures were asked about the reasons for non-participation (multiple choice items). One person responded, “The employees do not want to travel to the place where the measures are offered.” Three persons stated, “The employees do not have the time.” Another three stated that “The current measures do not fit the needs of our employees.” “Other” was chosen by seven people. Open statements regarding these “other” reasons were unfavorable appointments, the Corona pandemic, lack of knowledge, no information given to the employees, or regarding the offers and that the location did not fit. 

A further question focused on the basic possibility for employees’ participation. Here, 80% (*n* = 16 of 20) responded that it is rather likely to be possible, 20% (*n* = 4 of 20) that it is not. The persons who responded that it is rather not possible did not see an appropriate relationship between the effort and benefit of WHP, that the employees could not participate due to the high number of walk-in customers requiring their continuous presence, that the effort regarding travel to the measures is too high, or that the measures were communicated at the last minute (by the organizer).

To identify and understand the attitudes toward WHP offers, the managers or persons responsible for WHP were asked whether the companies had offered their employees improved opportunities to participate (multiple choice). In this regard, 17 out of 20 persons responded they allowed participation during working time, five out of 20 named “refund of traveling costs”, three answered that they were putting a car at the disposal of the employees, and another three persons responded that they had not done any of that.

### 3.4. Reasons for Non-Participation from the Employees’ Point of View

A KMO value of 0.65 and highly significant Bartlett test (*p* < 0.001) confirmed that an EFA can be performed. Five factors were extracted (Table 3). 

After a content check of the items, the fifth factor was excluded due to the lack of content-related cohesion of the items (“There wasn’t the right offer for me” and “I have the feeling that my colleagues wouldn’t approve”). As a result, a four-factor solution (Table 4) was suggested, and sum scores (mean) were calculated. 

The explained variance was 65.25%. The four factors found were labeled self-confidence, workload, endorsement, and need/interest. Regarding the mean values of each factor (see Table 5), it can be stated that workload is the factor with the highest agreement, and thus, the factor that can be seen as the greatest obstacle to participation in WHP measures from the employees’ point of view. The reliability measures (Table 5) can be rated as sufficient to good [45].

The performed Kendall rank correlations for testing the relationships between the four dimensions of non-participation and age of the employees showed no significant results (Table 5). For differences in sex (male/female as there were no reports on non-binary) the *t*-test and Mann–Whitney U-tests showed no significant results (Table 5).

## 4. Discussion

The present work focused on analyzing the flow of communication and information about WHP offers from company management to employees, plus barriers to participation. It can be stated that most employees knew about the offered measures and that the measures were cost free. The relationship between the communication of the measures to the employees and the idea that the WHP measures did not fit the needs of the companies’ workforce was not significant.

As second aim, the support of managers or persons responsible for WHP was investigated and the reasons for non-participation of the employees from the managers’ point of view were studied. It was found that most of the managers or persons responsible for WHP rated the measures as sufficiently varied and welcomed cross-network measures as well as allowed staff to take part in offers during working time. Reasons for non-participation from the managers’ point of view were the distance of the company to the location where the measures took place, lack of time, and a misfit between offers and employees’ needs.

From the employees’ point of view, workload (including working time) was the factor that was rated highest as a reason for non-participation. After that, self-confidence and need/interest were rated second place and at last the factor endorsement. No significant correlation between the factors for non-participation and age or sex were found.

It became clear that the managers or persons responsible for WHP allowed the employees to participate in the offers during their working time. At the same time, however, they highlighted that one of the reasons for non-participation from their point of view was the lack of time of the employees to participate in the offers. The employees also stated that missing time (factor workload) was the greatest obstacle (e.g., “I don’t have the time to participate in WHP measures” or “My workload doesn’t allow it”). This suggests that the opportunity aspect in the behavioral wheel [30] may not have been sufficiently addressed by the participating companies, especially regarding the investment of time. Implementing WHP can only be a resource in case it does not endanger existing demand–resource structures and their balance. Employees would need to perceive a very high individual benefit that outweighed the drawbacks [33]. When working schedules are already very full, including further tasks can quickly become a burden and becomes very improbable in case they are non-mandatory. Therefore, companies introducing WHP offers or providing the opportunity to use such via a network structure need to analyze the workload of staff together with further hindrances to participate in offers. 

Regarding the impression of discrepancies between offer and employee needs, further research is required as the offers were based on an employee survey. As the perceptions of WHP of the managers and employees might differ [46], the former might rate other offers as relevant, but the time lag between intervention design and offer together with a potential coverage error might also partly explain this result. 

### 4.1. Strenghts of the Studies

First, the studies carried out as part of the model project (article 20g SGB V PrävG) provide a substantial overview of the flow of information and communication regarding WHP in MSMEs in rather structurally weak regions. A total of two surveys were conducted, with special attention to the differences of the perspectives of the employees and the managers or those responsible for WHP. Obstacles to participation in WHP were also examined from the perspective of managers or persons responsible for WHP on the one hand and employees on the other. 

Second, there was a sufficient time lapse between the first offering of WHP measures and the second survey. Thus, all employees had had the opportunity to participate, and information diffusion could happen.

Third, it should be emphasized that the respective needs of the employees of the participating network companies were ascertained in advance as part of the legally required (psychological) risk assessments. It can, therefore, be assumed that the participating network companies had need for the WHP offers that were designed. 

Fourth, literature on WHP networks in MSMEs is scarce. Thus, the present analysis can provide further insights into this specific subarea.

### 4.2. Limitations of the Studies

The study variables were measured using leaders’ and employees’ self-reports. This is known as common method bias and might have led to responses showing social desirability. Due to this fact, participants were assured strict anonymity [47].

The results were obtained for the network companies of the model project GAIT. Therefore, both samples can not be considered representative of a larger population. We found no significant effects regarding the dimensions of non-participation, sex, and age as control variables (see Table 5). With a relatively small sample size, this could imply that effects that may be present in the population did not show up in our sample. 

The response rate of the first survey was 70% (managers and persons responsible for WHP) and 31.2% in the second survey (employees). While the response rate of the managers and persons responsible for WHP is high, those of the employees varies per network company and is moderate or even low. Some of the network companies are in the production sector without a fixed workstation. Here, a paper questionnaire might have been helpful to raise the response rates. In addition, workload might have been an issue, making any time spent away from official tasks problematic, even for replying to a survey. Despite the assured strict anonymity of the survey, some employees may have been reluctant to participate because the network companies are MSMEs and especially hesitant to provide open remarks due to fear of being recognizable.

As we combined two independent samples in our analyses, a clear match of the companies (regarding managers (first survey) and employees (second survey)) was not possible but is suggested for further research. However, studies among managers and employees on the reasons for non-participation in WHP measures state that a major barrier for participation is indeed time constraints [48,49,50].

The SARS-CoV-2 pandemic may have led to an additional impact on the reported results as the start of the on-site planned measures coincided with the beginning of the pandemic in March 2020. Participation in specific locations was, therefore, low, as managers or persons responsible for WHP and employees did not want to take any risks. Although the planned measures were quickly supplemented by corresponding online offers, the implementation of digital WHP measures is still not very routine. Technical equipment may have been lacking or digital fatigue due to the overload of online meetings, etc., might have led to lower participation. In addition, some may have used YouTube channels, etc., as substitute for network offers. 

Although an initial survey of employee WHM needs and requirements was conducted in all companies, the participation overlap with the evaluation survey reported here is unknown. However, the needs were very broad, which was reflected in the offers designed [40]. Thus, it can be assumed that most participants could find an offer corresponding to their wishes. Moreover, a lack of fit of the measures was not the main obstacle to participation, neither from the point of view of the managers or persons responsible for WHP nor from the point of view of the employees.

There are various individual factors that can influence participation in health-promotion offers, e.g., self-rated health or perceived risk of an illness [31,51], together with socioeconomic aspects (education, poverty [52,53,54]). As described above, the study covers perceived need for the offer, interest, and whether the offer did (not) fit the needs. We did not ask for the reasons for these feelings, for example, family history of an illness leading to perceived risk and, thus, interest in the offer, because WHP in Germany follows a salutogenic approach, which would discourage incorporating pathogenic messages in the program. However, the offers were based on the employees’ wishes and, thus, included their needs perceptions of needs, which may have served as proxies for individual motivations. 

### 4.3. Recommendations for Research and Practice

In the present work, companies in the service, trade, health and social services, and manufacturing sectors were involved. Depending on the place of work (display workstation or, e.g., shift work on the production line), the needs of employees may vary. Future research investigating participation barriers regarding job type is suggested. Moreover, future projects should link all surveys (initial needs survey and evaluation surveys of management and employees) and try to achieve high response rates. 

As mentioned above, the behavioral wheel is of specific relevance in our context. Behavior change is explained as depending on capability, motivation, and opportunity in the model [30]. Regarding opportunity, it can be stated that most people who took part in the employees’ survey knew about the offered measures. This indicates that there was probably just a minor lack of information and communication, but the opportunity to participate was given to the employees. For model projects or constellations in which the (potentially external) unit *organizing* the WHP measures passes on information about the offers to the managers and persons responsible for WHP, we suggest adding a further channel and communicating the offer also directly to the employees. Thus, reaching all staff members is ensured, even if there are internal communication disruptions. In addition, companies often want support in communicating and informing their employees, so providing content as well as helping to design structures is advisable. Future research could test the differences between projects that employ the dual communication strategies and those that do not, assuming the hypothesis that dual communication increases program recognition/knowledge about the offer and participation rates.

Ensuring a holistic approach in addition to WHP as a part of OSH, models should be developed in which the occupational physician (in some European Countries even mandatory by law for every company size [55]) can be involved more regularly and intensively in the whole process of WHP without having to devote additional resources. Future research could focus on analyzing the role of the occupational physician at the enterprise level [56] and at the same time whether this is desired by the companies or would be feasible.

As both groups mentioned the lack of time as an obstacle to participation in the offered WHP measures, we recommend viewing participation time as an investment in employee health. Healthy employees are a prerequisite for innovation, progress, and growth [1,2]. Thus, managers should focus on integrating setting-related prevention aimed at adapting the individual workload (including working time) of employees so that they can participate in the offered measures. Otherwise, WHP becomes an additional task that can be difficult to squeeze into a busy routine. To ensure that each person can participate in a WHP measure during a certain period, work plans must be designed accordingly. This should be implemented carefully to ensure that no inequities arise in the distribution of work. Companies wishing to offer WHP should first examine the feasibility of this project and subsequently select the appropriate implementation. If measures are not to be offered in the context of the needs of a single company but, e.g., for regional networks, the analyses and offers of measures have to take the potentially heterogeneous needs and locations of the participating companies into account. As reported here, regular meetings of the network companies to discuss locations for offers and ways to improve participation rates are highly recommended.

## 5. Conclusions

To the best of our knowledge, this is the first study to examine the attitudes toward WHP and to identify reasons for non-participation in WHP offers in a German model project according to article 20g SBG V PrävG. Using two independent studies carried out within the model project GAIT, we analyzed the flow of communication and information about WHP offers from company management to employees, investigated the support of managers or persons responsible for WHP and presented the reasons for non-participation of the employees from the managers’ point of view, as well as from the employees’ point of view. Special attention was paid to the differences of the perspectives of the employees and managers or persons responsible for WHP. Based on the results, recommendations for action to achieve higher participation rates in the future were given.

Future research should focus on matching datasets of employees and managers or persons responsible for WHP. This would generate more accurate insights, regarding the consensus of reasons for non-participation in WHP offers of both parties.

For the practical implementation of model projects in MSMEs, special attention should be paid to the aspects highlighted in the behavioral wheel. Based on our results, it is especially important to analyze the workload (including working time) and to focus on organizational interventions ensuring the opportunity to participate in the offers.

## Figures and Tables

**Table 1:** Participant characteristics.

Variable	First Survey Managers and Persons Responsible for WHP in the Network Companies (*n* = 21)	Second Survey Employees (*n* = 156)
**Age**	-	18–65 *M* = 40.2, *SD* = 11.5, *Md* = 39
**Sex**		
Male	15 (71.4%)	60 (38.5%)
Female	6 (28.6%)	91 (58.3%)
Non-binary	-	1 (0.6%)
**Job title/function**		
Executive director/top management	9 (42.9%)	3 (1.9%)
Supervisory function	5 (23.8%)	24 (15.4%)
Responsible for WHP	5 (23.8%)	-
Regular employee	-	127 (81.4%)
**Education**		
Not yet obtained a school-leaving qualification	-	1 (0.6%)
Certificate of secondary education (“Hauptschulabschluss”)	-	2 (1.3%)
General certificate of secondary education (“Realschulabschluss”)	-	71 (45.5%)
University entrance exam	-	33 (21.2%)
University degree	-	42 (26.9%)
**Working hours (per week)**		
>30	-	131 (84.0%)
Between 11 and 30	-	22 (14.1%)
<10 or exactly 10	-	1 (0.6%)
**Working time**		
Fixed and recorded	-	62 (39.7%)
Fixed and unrecorded	-	30 (19.2%)
Flexible	-	35 (22.4%)
Shift	-	18 (11.5%)
Trusted flextime	-	7 (4.5%)
**Place of work**		
Home office partly	-	46 (29.5%)
Home office completely	-	9 (5.8%)
On-site	-	99 (63.5%)

**Table 2:** Response rates.

Variable	First Survey	Second Survey	
	Managers and Persons Responsible for WHP in the Network Companies (*n* = 30)	By Companies (*n* = 30)	By Employees (*n* = 500)
Overall	21 (70.0%)	10 (33.3%)	156 (31.2%)
**Region**			
Central Thuringia	9 (30.0%)	5 (16.7%)	109 (21.8%)
South Thuringia	8 (26.7%)	4 (13.3%)	35 (7.0%)
East Thuringia	4 (13.3%)	1 (3.3%)	12 (2.4%)
**Company Size**			
Micro	-	1 (3.3%)	7 (1.4%)
Small	-	6 (20.0%)	68 (13.6%)
Medium	-	3 (10.0%)	81 (16.2%)

**Table 3:** Total variance explained by the five extracted factors (based on *n* = 156 questionnaires).

Factor	Initial Eigenvalues of Factors and Explained Variance			Rotated Sum of Squared Factor Loads and Explained Variance (after 5 Iterations) ^+^		
	Total Eigenvalue *	Explained Variance (%)	Cumulated Explained Variance (%)	Total Eigenvalue	Explained Variance (%)	Cumulated Explained Variance (%)
1	3.8	25.5	25.5	2.8	18.9	18.9
2	2.4	16.4	41.9	2.5	17.2	36.1
3	2.0	13.6	55.6	2.2	14.8	50.9
4	1.6	11.2	66.8	2.1	14.2	65.2
5	1.0	6.9	73.8	1.2	8.5	73.8

* Factors were extracted according to the Kaiser criterion (initial total eigenvalue > 1). ^+^ Rotation method: varimax.

**Table 4:** Assignment of individual items to underlying factors (reasons for non-participation).

Factor No. and Name	Item No.	Wording of the Item *	Factor Load	Mean
1. Self-confidence	1.1	I quickly feel exposed in groups.	0.82	3.40
	1.2	I don’t like to present results in front of others.	0.69	3.07
	1.3	I don’t like to be less fit or less trained than the other participants.	0.87	3.33
	1.4	I am afraid of embarrassing myself.	0.81	3.41
2. Workload	2.1	I don’t have the time to participate in WHP measures.	0.82	2.12
	2.2	Other issues have higher priority.	0.64	2.00
	2.3	My workload does not allow participation in WHP offers.	0.78	2.49
	2.4	Participation would only be possible outside working hours.	0.53	2.25
	2.5	It is not easy to integrate WHP offers into my work routine (e.g., shift work, customer contact).	0.67	2.70
3. Endorsement	3.1	I have the feeling that my supervisor would not approve.	0.89	3.63
	3.2	I have the feeling that my employer does not approve.	0.90	3.72
4. Need/Interest	4.1	I have no need for WHP measures.	0.91	3.19
	4.2	I am generally not interested in participating in WHP measures.	0.84	3.41

Abbreviations: WHP—workplace health promotion. * Answer options: applies (1), does rather apply (2), does rather not apply (3), does not apply (4).

**Table 5:** Means, Cronbach’s alpha, and *p*-values of the dimensions of non-participation.

	Mean (*M*) *	Cronbach’s Alpha (α)	Age (*p*-Value)	Sex (*p*-Value)
Self-confidence	3.3	0.88	0.62	0.13
Workload	2.2	0.62	0.61	0.42
Endorsement	3.7	0.84	0.48	0.20
Need/Interest	3.3	0.80	0.44	0.20

* Answer options: applies (1), does rather apply (2), does rather not apply (3), does not apply (4).

## Data Availability

The datasets analyzed during the current study are not publicly available due to German national data protection regulations but are available from the corresponding author on reasonable request.

## References

[1] Hager F. (2018). Gender and Leadership? Do female leaders perform a different, better or even healthier Leadership Style?. Int. Inst. Soc. Econ. Sci..

[2] Pundt F., Felfe J. (2017). HOL. An Instrument to Assess Health-Oriented Leadership, Göttingen. https://www.testzentrale.de/shop/health-oriented-leadership.html.

[3] Ferreira Y., Vogt J. (2021). Psychische Belastung und deren Herausforderungen. Z. Für Arb..

[4] Costa G. (2010). Shift work and health: Current problems and preventive actions. Saf. Health Work.

[5] Theorell T., Hammarström A., Aronsson G., Träskman Bendz L., Grape T., Hogstedt C., Marteinsdottir I., Skoog I., Hall C. (2015). A systematic review including meta-analysis of work environment and depressive symptoms. BMC Public Health.

[6] Bryson A., Forth J., Stokes L. (2015). Does Worker Wellbeing Affect Workplace Performance?. SSRN J..

[7] Pieper C., Schröer S., Eilerts A.-L. (2019). Evidence of Workplace Interventions-A Systematic Review of Systematic Reviews. Int. J. Environ. Res. Public Health.

[8] (2021). Die Techniker. Gesundheitsreport 2021-Arbeitsunfähigkeiten, Hamburg. https://www.tk.de/resource/blob/2110096/11c10b8be736a0f2b70e40c01cadba63/2021-tk-gesundheitsreport-data.pdf.

[9] Grobe T.G., Braun A. (2021). BARMER Gesundheitsreport 2021; Schriftenreihe zur Gesundheitsanalyse No. 31, Berlin. https://www.barmer.de/resource/blob/1032110/aaafa3405427f0b05d34a7f20fd904d1/barmer-gesundheitsreport-2021-data.pdf.

[10] Marschall J., Hildebrandt S., Gerb J., Nolting H.-D. (2021). Gesundheitsreport 2021: Coronakrise und Digitalisierung, Hamburg. https://www.dak.de/dak/download/report-2515312.pdf.

[11] Statista. Volkswirtschaftliche Produktionsausfallkosten Aufgrund von Arbeitsunfähigkeit in Deutschland nach Diagnosegruppe im Jahr 2020. https://de.statista.com/statistik/daten/studie/869779/umfrage/produktionsausfallkosten-aufgrund-von-arbeitsunfaehigkeit-in-deutschland-nach-diagnose/.

[12] Bundesministerium für Arbeit und Soziales (2021). Sicherheit und Gesundheit bei der Arbeit–Berichtsjahr 2020.

[13] Dannheim I., Ludwig-Walz H., Buyken A.E., Grimm V., Kroke A. (2021). Effectiveness of health-oriented leadership interventions for improving health and wellbeing of employees: A systematic review. J. Public Health.

[14] Franke F., Felfe J. (2011). How does transformational leadership impact employees’ psychological strain?. Leadership.

[15] Gregersen S., Kuhnert S., Zimber A., Nienhaus A. (2011). Führungsverhalten und Gesundheit-Zum Stand der Forschung. Gesundheitswesen.

[16] Jimenez P., Winkler B., Dunkl A. (2017). Creating a healthy working environment with leadership: The concept of health-promoting leadership. Int. J. Hum. Resour. Manag..

[17] Bregenzer A., Milfelner B., Šarotar Žižek S., Jiménez P. (2020). Health-Promoting Leadership and Leaders’ Listening Skills Have an Impact on the Employees’ Job Satisfaction and Turnover Intention. Int. J. Bus. Commun..

[18] Faller G. (2018). Umsetzung Betrieblicher Gesundheitsförderung/Betrieblichen Gesundheitsmanagements in Deutschland: Stand und Entwicklungsbedarfe der einschlägigen Forschung. Gesundheitswesen.

[19] Badura B., Ritter W., Scherf M. (1999). Betriebliches Gesundheitsmanagement-ein Leitfaden für die Praxis.

[20] Drexler H., Letzel S., Nesseler T., Stork J., Tautz A. Arbeitsmedizin 4.0, Thesen der Arbeitsmedizin zum Stand und zum Entwicklungsbedarf der Betrieblichen Prävention und Gesundheitsförderung in Deutschland: Stellungnahme der Deutschen Gesellschaft für Arbeitsmedizin und Umweltmedizin. https://www.dgaum.de/fileadmin/pdf/Artikel/ASU_2015-10_Arbeitsmedizin_4.0_Broschuere_final.pdf.

[21] Schill A.L., Chosewood L.C. (2013). The NIOSH Total Worker Health™ program: An overview. J. Occup. Environ. Med..

[22] Bundeministerium für Gesundheit. Das Präventionsgesetz. https://www.bundesgesundheitsministerium.de/service/begriffe-von-a-z/p/praeventionsgesetz.html.

[23] Mazzola J.J., Jackson A.T., Thiele A. (2019). Obesity in the Workplace: A Systematic Review of Barriers and Facilitators to Healthy Lifestyles. Occup. Health Sci..

[24] Kwak L., Kremers S.P.J., van Baak M.A., Brug J. (2006). Participation rates in worksite-based intervention studies: Health promotion context as a crucial quality criterion. Health Promot. Int..

[25] Reinhardt A., Adams J., Schöne K., Rose D.-M., Sammito S. (2020). Do working characteristics influence the participation at health measures? Findings from a trial phase of workplace health promotion. J. Occup. Med. Toxicol..

[26] Lier L.M., Breuer C., Dallmeyer S. (2019). Organizational-level determinants of participation in workplace health promotion programs: A cross-company study. BMC Public Health.

[27] Kilpatrick M., Blizzard L., Sanderson K., Teale B., Jose K., Venn A. (2017). Barriers and facilitators to participation in workplace health promotion (WHP) activities: Results from a cross-sectional survey of public-sector employees in Tasmania, Australia. Health Promot. J. Austr..

[28] Perrault E.K., Hildenbrand G.M., Rnoh R.H. (2020). Employees’ Refusals to Participate in an Employer-Sponsored Wellness Program: Barriers and Benefits to Engagement. Compens. Benefits Rev..

[29] Nöhammer E., Stummer H., Schusterschitz C. (2014). Employee perceived barriers to participation in worksite health promotion. J. Public Health.

[30] Michie S., van Stralen M.M., West R. (2011). The behaviour change wheel: A new method for characterising and designing behaviour change interventions. Implement. Sci.

[31] Toker S., Heaney C.A., Ein-Gar D. (2015). Why won’t they participate? Barriers to participation in worksite health promotion programmes. Eur. J. Work. Organ. Psychol..

[32] Dauner K.N., McIntosh C.R., Xiu L. (2019). Determinants of workplace health program participation among non, low, and incentive-achieving participants. J. Workplace Behav. Health.

[33] Nöhammer E. (2022). Designing attractive workplace health promotion programs. Empl. Relat. Int. J..

[34] Lösch R., Amler N., Drexler H. (2022). Arbeits- und Gesundheitsschutz und Betriebliches Eingliederungsmanagement in Deutschland–Ein systematisches Review zum Umsetzungsstand gesetzlicher Vorgaben. Gesundheitswesen.

[35] Hollederer A., Wießner F. (2015). Prevalence and development of workplace health promotion in Germany: Results of the IAB Establishment Panel 2012. Int. Arch. Occup. Environ. Health.

[36] Ansmann L., Jung J., Nitzsche A., Pfaff H. (2012). Zusammenhänge zwischen der Betriebsstruktur und Betrieblichem Gesundheitsmanagement in der Informationstechnologie- und Kommunikationsbranche. Gesundheitswesen.

[37] Beck D., Lenhardt U., Schmitt B., Sommer S. (2015). Patterns and predictors of workplace health promotion: Cross-sectional findings from a company survey in Germany. BMC Public Health.

[38] Schaefer E., Drexler H., Kiesel J. (2016). Betriebliche Gesundheitsförderung in kleinen, mittleren und großen Unternehmen des Gesundheitssektors-Häufigkeit, Handlungsgründe der Unternehmensleitungen und Hürden der Realisierung. Gesundheitswesen.

[39] Hoffmann C., Stassen G., Schaller A. (2020). Theory-Based, Participatory Development of a Cross-Company Network Promoting Physical Activity in Germany: A Mixed-Methods Approach. Int. J. Environ. Res. Public Health.

[40] Lösch R., Fischmann W., Drexler H. (2020). Passgenaues betriebliches Gesundheitsmanagement–nichts leichter als das?. ASU.

[41] Stummer H., Nöhammer E., Schaffenrath-Resi M., Eitzinger C. (2008). Interne Kommunikation und betriebliche Gesundheitsförderung. Praev. Gesundheitsf..

[42] Tabachnick B.G., Fidell L.S. (2014). Using Multivariate Statistics.

[43] Hair J.F., Black W.C., Babin B.J., Anderson R.E. (2019). Multivariate Data Analysis.

[44] Pituch K.A., Stevens J.P. (2016). Applied Multivariate Statistics for the Social Sciences: Analyses with SAS and IBM’s SPSS.

[45] Schermelleh-Engel K., Werner C.S., Moosbrugger H., Kelava A. (2012). Methoden der Reliabilitätsbestimmung. Testtheorie und Fragebogenkonstruktion.

[46] McCleary K., Goetzel R.Z., Roemer E.C., Berko J., Kent K., La Torre H.D. (2017). Employer and Employee Opinions About Workplace Health Promotion (Wellness) Programs: Results of the 2015 Harris Poll Nielsen Survey. J. Occup. Environ. Med..

[47] Podsakoff P.M., MacKenzie S.B., Lee J.-Y., Podsakoff N.P. (2003). Common method biases in behavioral research: A critical review of the literature and recommended remedies. J. Appl. Psychol..

[48] Sargent G.M., Banwell C., Strazdins L., Dixon J. (2018). Time and participation in workplace health promotion: Australian qualitative study. Health Promot. Int..

[49] Stiehl E., Bales S.L., Jenkins K.R., Sherman B.W. (2022). Unique Barriers to Workplace Health Promotion Programs by Wage Category: A Qualitative Assessment of Secondary Data. Am. J. Health Promot..

[50] Kim M., Lin Y.C., Luna G., Ma J., Stiehl E. (2020). Certified Nursing Assistants’ Barriers and Facilitators to Accessing and Using Worksite Health Promotion Programs. J. Occup. Environ. Med..

[51] Niessen M.A.J., Laan E.L., Robroek S.J.W., Essink-Bot M.-L., Peek N., Kraaijenhagen R.A., van Kalken C.K., Burdorf A. (2013). Determinants of Participation in a Web-Based Health Risk Assessment and Consequences for Health Promotion Programs. J. Med. Internet Res..

[52] van Heijster H., Boot C.R.L., Robroek S.J.W., Oude Hengel K., van Berkel J., de Vet E., Coenen P. (2021). The effectiveness of workplace health promotion programs on self-perceived health of employees with a low socioeconomic position: An individual participant data meta-analysis. SSM-Popul. Health.

[53] Côté M., Harrison S., Lapointe A., Laramée C., Desroches S., Lemieux S., Lamarche B., Bélanger-Gravel A. (2020). A cross-sectional survey examining motivation and beliefs to participating in a web-based prospective cohort study on nutrition and health among individuals with a low socioeconomic status. BMC Public Health.

[54] Stiehl E., Shivaprakash N., Thatcher E., Ornelas I.J., Kneipp S., Baron S.L., Muramatsu N. (2018). Worksite Health Promotion for Low-Wage Workers: A Scoping Literature Review. Am. J. Health Promot..

[55] WHO European Centre for Environment and Health (2000). Occupational Medicine in Europe: Scope and Competencies.

[56] Schubin K., Schlomann L., Lindert L., Pfaff H., Choi K.-E. (2020). Occupational Physicians’ Perspectives on Determinants of Employee Participation in a Randomized Controlled Musculoskeletal Health Promotion Measure: A Qualitative Study. Int. J. Environ. Res. Public Health.

